# Disparity of perspectives between teachers and learners on perioperative teaching and learning

**DOI:** 10.1186/s12909-020-02172-8

**Published:** 2020-07-31

**Authors:** Yu-Tang Chang, Peih-Ying Lu, Chung-Sheng Lai

**Affiliations:** 1 Department of Surgery, Kaohsiung Medical University Hospital, Kaohsiung, Taiwan; 2 School of Medicine, College of Medicine, Kaohsiung Medical University, Kaohsiung, Taiwan; 3 College of Humanities and Social Sciences, Kaohsiung Medical University, Kaohsiung, Taiwan

**Keywords:** Perioperative teaching and learning, Operating room, Perspectives, Learning objectives, Working hours

## Abstract

**Background:**

To build a consensus about learning objectives in the operating room, the aim of the study was to evaluate both surgical teacher and learner perspectives on perioperative teaching and learning in Taiwan.

**Methods:**

Twelve main technical and non-technical learning objectives in the operating room were evaluated by learners and surgical teachers in Kaohsiung Medical University Hospital. The learners included postgraduate year (PGY) 1–3 residents (junior learner, JL) and PGY 4–7 residents (senior learner, SL). The definition of learning preferences were recommended learning objectives, and learning load was defined as demands of learning preferences. During the survey, surgical teachers evaluated the learning preferences for the learner, and learners evaluated their learning preferences. The learners also evaluated the learning preferences that the surgical teachers should teach.

**Results:**

Response rate of the questionnaire was 65.4%. A total of 31 learners and 39 surgical teachers completed the survey. The consensus was that the need to increase the learning loads and ethical issues were the learning preferences for SL, and indications, details of procedure, and teamwork were important to both JL and SL. The teachers intended to set specific learning objectives for different learner levels, including (i) indications, details of procedure, teamwork, and postoperative care for both JL and SL; (ii) preoperative preparation, surgical anatomy, and instrument handling for JL (*P* = 0.022, 0.021 and 0.006); and (iii) surgical technique, independent practice, clinical reasoning, complications, and ethical issues for SL (*P* = 0.010, < 0.001, < 0.001, 0.001, 0.011). Resident perspective on learning objectives differed between JL and SL, and there was discrepancy between resident’s learning as perceived by teachers, particularly in the JL.

**Conclusions:**

Our study revealed significant disparity of perspectives between teachers and learners on perioperative teaching and learning. Surgical teachers should set specific learning objectives for different learner levels, since junior and senior residents have different learning preferences even though both scrub in the same case. Effective communication between teachers and learners has the potential to improve learning experience and create a positive environment in the operating room.

## Background

The apprenticeship model for surgical training has been a long-standing gold standard worldwide, with significant time demands of learners working under supervision until being judged competent to operate on their own [1, 2]. However, long working hours might increase the risk of making medical errors [3]. Therefore, duty hours of the residents in the United States have been restricted by the Accreditation Council for Graduate Medical Education Common Program Requirements since 2003 [4]; similar standards were not implemented in Taiwan until 2013. In March 2017, the Ministry of Health and Welfare of Taiwan announced working-hour guidelines for resident physicians where maximum weekly working hours were reduced from 88 to 80 [5]. Starting from September 2019, residents were incorporated into the National Labor Standards Act. At present, residents in Taiwan have fixed work hours and work under a responsibility system [6]. With more restrictions on working hours, the working enviro nment for resident physicians and patient safety can be improved [4]; however, due to limited time in the hospital, it is necessary to improve the efficiency of surgical training for resident physicians [7–9].

The importance of intraoperative teaching is coming into great focus [2, 10–14]. Several interventions and principles of practice have been developed to improve intraoperative education [7, 12, 15, 16]. The briefing, intraoperative teaching, debriefing “BID” model reported by Roberts et al. identified that the teacher should “brief” the learner before the case, then teach in the operating room, and then “debrief” about what goals were met and how learners can improve for the next case [15]; however, there is significant discrepancy between resident and surgical teacher perspectives on preoperative preparation as well as intraoperative teaching and postoperative feedback [17–20]. To improve the learning environment in the operating room, the learners and the teachers both need to build a consensus about learning and teaching objectives. In Taiwan, few studies have demonstrated the resident and surgical teacher perspectives in the operating room [8]; accordingly, the purpose of this study was to evaluate learner and teacher perspectives on learning and teaching objectives in the operating room.

## Methods

### Study setting

In Taiwan, the residency training program has followed the similar program of the American Medical Association since 1950 [21]. Medical students graduate from medical school after completing 6 years of college education and 1 year of internship training and then chose their specialty in the residency training program. In 2003, a 3-month post-graduation general medicine training program was implemented throughout Taiwan, and 1 year of postgraduate general medicine training was introduced [21]. Since 2013, the medical education program has changed from 7 years to 6 years, moving the last year internship to postgraduate year (PGY) 1 resident that is like the training systems in Japan and in the United Kingdom [22, 23].

The Institutional Review Board at Kaohsiung Medical University Hospital approved this study (KMUHIRB-E(II)-20,180,262). Participation in the survey was voluntary and without compensation. Teachers were attending surgeons from various surgical specialties, whereas learners included PGY 1–3 residents (junior learner, JL), and PGY 4–7 residents (senior learner, SL) [19]. Participant surgeons and learners provided written informed consent and completed the survey.

### Survey instrument

We have referred to the resident performance assessments in the American Broad Surgery (ABS) and Non-Technical Skills for Surgeons (NOTSS) behavior marker system defined by the Royal College of Surgeons of Edinburgh when creating our survey instrument [24, 25]. The assessment of operative and clinical performance in the ABS standardizes the knowledge and skills (e.g. steps of procedure and independent practice) expected of general surgery residents, while the NOTSS behavior marker system was developed to evaluate learner non-technical performance. Then we had a panel of experts reach consensus regarding what technical and non-technical skills to be included. We also reviewed related literature to construct detailed learning objectives. Agha et al. has emphasized that non-technical skills such as communication and teamwork skills and decision-making are of increasing importance in surgery and surgical training [9]. Rose et al. reported that technical skills such as operative steps, surgical skills, instrument handling, and surgical technique were significantly different between surgical residents and faculty [17]. Furthermore, local context was considered, and Lai et al. have reported that teaching of clinical reasoning in surgery is of paramount importance to stimulate learner’s self-directed learning [8, 26]. To evaluate the complete educational activities in the operating room, learning objectives before and after surgery were also included. Therefore, 12 specific technical and non-technical learning objectives, including preoperative preparation, surgical anatomy, operative indications, instrument handling, surgical technique, details of procedure, independent practice, clinical reasoning, complications, teamwork, ethical issues and postoperative care were evaluated using a standardized questionnaire.

The definition of perioperative teaching was expected objectives provided by surgical teachers, while the definition of perioperative learning was objectives that residents desired to learn. Among twelve main technical and non-technical learning objectives, preoperative preparation was defined as the preparation before the surgery in the operating room such as aseptic environment, temperature and patient position. Details of procedure referred to explicit explanation and verbally describing surgical procedures step-by-step. Ethical issues in surgery were defined as medical errors, truth-telling, and disclosure [27]. Postoperative care was defined as the care after surgery in the operating room such as evaluation of skin integrity, patient transportation and immediate operative reports. The definition of learning preference included technical and non-technical skills that were recommended to be learnt, and learning load was defined as demands of learning preferences.

### Data collection

During the survey, surgical teachers evaluated the learning preferences for the learner (Group I, teacher perspective on learning), and learners evaluated their learning preferences (Group II, learner perspective on learning). The learners also evaluated the learning preferences that the surgical teachers should teach (Group III, learner perspective on teaching). More detailed information on the performance characteristics of the survey instruments, including the original Chinese-language survey, and English-language version, are provided in the Additional file 1.

Surgical teachers were analyzed for their demographic variables (age at evaluation, gender, seniority as an attending surgeon, seniority as a faculty member of the medical school, experience of annual teaching award, and percentage of effort teaching). Secondly, 12 selected objectives were evaluated among the groups. The perspectives between junior and senior learners were also compared.

### Statistical analysis

Student *t*-tests and χ2 tests were used to compare continuous and categorical descriptive variables respectively between learners and surgical teachers. The differences in enrolled learners and surgical teachers were compared across three groups using analysis of variance (ANOVA) for continuous variables and χ^2^ tests for categorical variables. Further analysis with Scheffé post-hoc test was planned when the ANOVA showed that there was a statistical difference between groups. A *P* value less than 0.05 denoted statistical significance. SPSS for Windows version 20.0 was used for all statistics.

## Results

### Demographic data

In September 2018, a total of 65 surgical teachers and 42 learners were surveyed. We collected responses from 31 learners and 39 surgical teachers (response rate: 65.4%). The 31 learners included two PGY1 residents, 11 PGY2 residents, six PGY3 residents, three PGY4 residents, five PGY5 residents, three PGY6 residents and one PGY7 resident. Overall, there were 19 JL and 12 SL.

The characteristics of participating surgical teachers are shown in Table 1. The average seniority as an attending surgeon was 14.9 ± 10.9 years, ranging from 1 to 40 years. Of 21 surgeons who had faculty positions at the medical school, one was a lecturer, eight were assistant professors, four were associate professors, and eight were full professors. Most of the teachers (56.4%) responded that teaching constituted between 21 and 40% of their overall efforts. Eighteen surgeons indicated they had received at least one teaching award during their career. Among 39 surgical teachers, there were 29 (74.4%) general surgeons, 4 (10.2%) plastic surgeons and 6 (15.4%) neurosurgeons. There were no statistically significant differences in age, seniority as an attending surgeon, seniority as a faculty member of the medical school, and experience of annual teaching award among three specialties (*P* = 0.357, 0.453, 0.803 and 0.415).

**Table 1:** Characteristics of surgical teachers

Surgical teachers (*N* = 39)	Items	N	%
Gender	Male	36	92.3
	Female	3	7.7
Age	31–50 years	23	59.0
	> 50 years	16	41.0
Seniority as an attending	< 15 years	19	48.7
	≥15 years	20	51.3
Faculty member of medical school	No	18	46.2
	Yes	21	53.8
Effort teaching (%)	0–20%	8	20.5
	21–40%	22	56.4
	> 41%	9	23.1

### Comparison between junior learners and senior learners

#### Teacher perspective on learning (group I)

The surgical teachers perceived that SL should require more learning objectives (8.9 ± 3.0 vs 7.8 ± 2.5; *P* = 0.049). Instrument handling (84.6%) was the most important learning objective for JL, while independent practice (94.9%) was indicated for SL. In comparison between JL and SL, preoperative preparation, surgical anatomy and instrument handling were indicated as preferences for JL (*P* = 0.022, 0.021, and 0.006), while surgical technique, independent practice, clinical reasoning, complications and ethical issues were indicated for SL (*P* = 0.010, < 0.001, < 0.001, 0.001, and 0.011). Details of procedure and teamwork were recommended learning preferences for both JL and SL (Table 2).

**Table 2:** Teacher and learner perspectives in the operating room

Learning objectives	Group I Teacher perspective on learning		*P*	Group II Learner perspective on learning		*P*	Group III Learner perspective on teaching		*P*
	JL N (%)	SL N (%)		JL N (%)	SL N (%)		JL N (%)	SL N (%)	
Number of learning objectives	7.8 ± 2.5	8.9 ± 3.0	0.049	6.3 ± 3.5	8.3 ± 3.5	0.795	4.4 ± 2.4	6.8 ± 4.1	0.007
Preoperative preparation	27 (69.2)	17 (43.6)	0.022	7 (36.8)	7 (58.3)	0.242	6 (31.6)	6 (50.0)	0.305
Surgical anatomy	28 (71.8)	18 (46.2)	0.021	14 (73.7)	11 (91.7)	0.132	12 (63.2)	9 (75.0)	0.492
Indications	32 (82.1)	25 (64.1)	0.074	13 (68.4)	11 (91.7)	0.217	12 (63.2)	8 (66.7)	0.842
Instrument handling	33 (84.6)	22 (56.4)	0.006	7 (36.8)	6 (50.0)	0.470	2 (10.5)	4 (33.3)	0.117
Surgical technique	27 (69.2)	36 (92.3)	0.010	16 (84.2)	10 (83.3)	0.948	10 (52.6)	9 (75.0)	0.213
Details of procedure	32 (82.1)	32 (82.1)	1.000	14 (73.7)	8 (66.7)	0.675	9 (47.4)	6 (50.0)	0.886
Independent practice	12 (30.8)	37 (94.9)	< 0.001	13 (68.4)	10 (83.3)	0.355	6 (31.6)	9 (75.0)	0.018
Clinical reasoning	18 (46.2)	36 (92.3)	< 0.001	9 (47.4)	6 (50.0)	0.886	6 (31.6)	6 (50.0)	0.305
Complications	24 (61.6)	36 (92.3)	0.001	9 (47.4)	8 (66.7)	0.293	6 (31.6)	8 (66.7)	0.056
Teamwork	25 (64.1)	28 (71.8)	0.467	6 (31.6)	5 (38.5)	0.567	5 (26.3)	5 (35.8)	0.373
Ethical issues	18 (46.2)	29 (74.4)	0.011	5 (26.3)	8 (66.7)	0.027	2 (10.5)	6 (50.0)	0.014
Postoperative care	26 (66.7)	29 (74.4)	0.456	7 (36.8)	9 (75.0)	0.038	8 (42.1)	6 (50.0)	0.667

*JL* Junior learner, *SL* Senior learner

#### Learner perspective on learning (group II)

JL perceived that surgical technique (84.2%) was the most important learning objective; meanwhile, both surgical anatomy and indications (91.7%) were indicated for SL. In comparison between JL and SL, ethical issues and postoperative care were the SL’s learning preferences (*P* = 0.027 and 0.038) (Table 2).

#### Learner perspective on teaching (group III)

The learners perceived that surgical teachers should teach more objectives to SL (6.8 ± 4.1 vs 4.4 ± 2.4; *P* = 0.007). JL perceived that surgical anatomy (63.2%) and indications (63.2%) were the learning objectives that surgical teacher should provide; meanwhile, SL perceived that surgical anatomy (75%), surgical technique (75%) and independent practice (75%) were the objectives. In comparison between JL and SL, independent practice and ethical issues were the SL’s preferences that the teachers should teach (*P* = 0.018 and 0.014) (Table 2).

#### Surgical teacher and learners perspectives among the three groups

The teachers, JL and SL shared common ground when discussing indications, details of procedure, teamwork, and ethical issues, with the consensus being that ethical issues were the learning preference for SL, whereas indications, details of procedure, and teamwork were important to both JL and SL. However, resident perspective on learning objectives differed between JL and SL, and there was discrepancy between resident’s learning as perceived by teachers, particularly in the JL (Table 2).

### Comparison of surgical teacher and learner perspectives on learning (group I vs. group II)

The demands of the learning objectives between surgical teacher and learner preferences were compared. For JL, the number of the learning objectives from the surgical teacher perspective was more than those from the learner perspective (*P* = 0.042); however, there was no significant difference for SL. Of the 12 objectives, JL responses differed from teacher responses on five objectives and SL responses on three. From teacher perspective, preoperative preparation, instrument handling, teamwork and postoperative care were the preferences for the JL (*P* = 0.019, < 0.001, 0.020 and 0.031); however, the JL’ s preference was independent practice (*P* = 0.007). SL saw surgical anatomy was the learning preference (*P* = 0.005); whereas clinical reasoning and complications were the preferences for SL from the teacher perspective (*P* = 0.001, and 0.024) (Table 3).

**Table 3:** Comparison of learner and teacher perspectives on learning (Group I vs. Group II) in the operating room

Learning objectives	Junior learner		*P*	Senior learner		*P*
	Learner perspective	Teacher perspective		Learner perspective	Teacher perspective	
	*N* = 19	*N* = 39		*N* = 12	*N* = 39	
Number of learning objectives	6.3 ± 3.5	7.8 ± 2.5	0.042	8.3 ± 3.5	8.9 ± 3.0	0.334
Preoperative preparation	7 (36.8)	27 (69.2)	0.019	7 (58.3)	17 (43.6)	0.371
Surgical anatomy	14 (73.7)	28 (71.8)	0.880	11 (91.7)	18 (46.2)	0.005
Indications	13 (68.4)	32 (82.1)	0.243	11 (91.7)	25 (64.1)	0.067
Instrument handling	7 (36.8)	33 (84.6)	< 0.001	6 (50.0)	22 (56.4)	0.696
Surgical technique	16 (84.2)	27 (69.2)	0.221	10 (83.3)	36 (92.3)	0.361
Details of procedure	14 (73.7)	32 (82.1)	0.460	8 (66.7)	32 (82.1)	0.257
Independent practice	13 (68.4)	12 (30.8)	0.007	10 (83.3)	37 (94.9)	0.194
Clinical reasoning	9 (47.4)	18 (46.2)	0.931	6 (50.0)	36 (92.3)	0.001
Complications	9 (47.4)	24 (61.6)	0.306	8 (66.7)	36 (92.3)	0.024
Teamwork	6 (31.6)	25 (64.1)	0.020	5 (38.5)	28 (71.8)	0.056
Ethical issues	5 (26.3)	18 (46.2)	0.147	8 (66.7)	29 (74.4)	0.602
Postoperative care	7 (36.8)	26 (66.7)	0.031	9 (75.0)	29 (74.4)	0.964

### Comparison between teacher perspective on learning and learner perspective on teaching (group I vs. group III)

Learner and teacher perspectives on learning loads were compared. The surgical teacher perceived that JL required more learning objectives (7.8 ± 2.5) compared with the JL perspective (4.4 ± 2.4) (*P* <  0.001), but there was no significant difference for the SL perspective (*P* = 0.065). Of the 12 objectives, the disparity of perspectives between JL and teachers was six, including preoperative preparation, instrument handing, details of procedure, complications, teamwork and ethical issues (*P* = 0.007, < 0.001, 0.006, 0.032, 0.007 and 0.007). However, SL responses differed from teacher responses on four objectives, including details of procedure, independent practice, clinical reasoning and complications (*P* = 0.026, 0.043, 0.001 and 0.024).

The surgical teachers perceived that preoperative preparation, instrument handling, details of procedure, complications, teamwork and ethical issues were the preferences for JL (*P* = 0.007, < 0.001, 0.006, 0.032, 0.007 and 0.007), and details of procedure, independent practice, clinical reasoning and complications were the preferences for SL (*P* = 0.026, 0.043, 0.001, and 0.024) (Table 4).

**Table 4:** Comparison between teacher perspective on learning (Group I) and learner perspective on teaching (Group III) in the operating room

Learning objectives	Junior learner		*P*	Senior learner		*P*
	Teacher perspective on learning	Learner perspective on teaching		Teacher perspective on learning	Learner perspective on teaching	
	*N* = 39	*N* = 19		*N* = 39	*N* = 12	
Number of learning objectives	7.8 ± 2.5	4.4 ± 2.4	< 0.001	8.9 ± 3.0	6.8 ± 4.1	0.065
Preoperative preparation	27 (69.2)	6 (31.6)	0.007	17 (43.6)	6 (50.0)	0.696
Surgical anatomy	28 (71.8)	12 (63.2)	0.505	18 (46.2)	9 (75.0)	0.080
Indications	32 (82.1)	12 (63.2)	0.115	25 (64.1)	8 (66.7)	0.871
Instrument handling	33 (84.6)	2 (10.5)	< 0.001	22 (56.4)	4 (33.3)	0.162
Surgical technique	27 (69.2)	10 (52.6)	0.217	36 (92.3)	9 (75.0)	0.104
Details of procedure	32 (82.1)	9 (47.4)	0.006	32 (82.1)	6 (50.0)	0.026
Independent practice	12 (30.8)	6 (31.6)	0.950	37 (94.9)	9 (75.0)	0.043
Clinical reasoning	18 (46.2)	6 (31.6)	0.290	36 (92.3)	6 (50.0)	0.001
Complications	24 (61.6)	6 (31.6)	0.032	36 (92.3)	8 (66.7)	0.024
Teamwork	25 (64.1)	5 (26.3)	0.007	28 (71.8)	5 (35.8)	0.056
Ethical issues	18 (46.2)	2 (10.5)	0.007	29 (74.4)	6 (50.0)	0.112
Postoperative care	26 (66.7)	8 (42.1)	0.075	29 (74.4)	6 (50.0)	0.112

## Discussion

Due to gradually increasing medical disputes and legal liability, the relationship between physicians and patients has become tense [28]. As a result, physicians have started to choose defensive medicine practice, and the brain drain in high-risk specialties, especially for surgeons, has continued unabated [28, 29]. From 2006 through 2018, according to the Taiwan Medical Association, the number of member physicians has increased from 35,936 to 49,019 (2.8% increase per year) and the number of board-certified surgeons from 3027 to 3830 (2.0% increase per year) [30]. For every 1000 new physicians, only 61 choose surgery as a career although there are more than 300 positions of surgical residency provided for 1500 postgraduates each year in Taiwan. The dwindling interest in surgery and surgical specialties raises concerns that surgical manpower requirements might reach a deficit in coming years [31]. In 2014, the National Health Research Institutes of Taiwan reported that there will be more than 1500 vacancies of surgical specialty in Taiwan after 2022 [32]. Factors that disaffect young doctors in choosing high-risk specialties such as surgery are long duty hours, increased legal liability, and low payments from the National Health Insurance System [32]. The restriction on duty hours is the policy to improve the working environment for resident physicians. However, the teaching hospital is normally concerned that work hour restrictions will limit clinical training time and even worsen the decrease of working hours. In the era of competency-based surgical training, how to create a more effective learning environment has become key to improve surgical education and enhance junior doctors’ confidence in pursuing a career in surgery [29].

For the training program of surgical residency, the operating room is the main educational theater. However, our study identified different learning preferences among teachers, junior learners, and senior learners. The educational activity in the operating room cannot be efficient if teachers and learners do not agree on what should be taught. Lack of communication between learners and teachers might account for significant discrepancy between resident and surgical teacher perspectives [2]. Communication barriers in the operating room would impede learning objectives from being efficiently delivered. However, the relationship involves both sides. For example: teachers may stay silent or keep blaming during the operation, while learners would fear or hesitate to express their expectations. If neither teachers nor learners leverage the communication, it might be difficult for learners to get the most out of their education. The solution to the problem would be for teachers to decide what they will teach at the different learner levels. Effective communication between teachers and learners has the potential to improve the learning experience and create a positive environment in the operating room.

In the past, the volume of surgical procedures was the key to surgical training in the operating room [15]. However, owing to limited duty hours of the residency and the increasing volume of surgical procedures, it is impossible for learners to learn at one time. Unguided (pure) discovery learning is ineffective and inefficient [15]. A good model for deliberate teaching in the operating room would allow the teacher to focus more on setting objectives for the learner’s performance, providing immediate and specific feedback, and providing guidance for future practice [15]. Agha et al. recommended that modern surgical practice requires technical and non-technical skills, evidence-based practice, an emphasis on lifelong learning, monitoring of outcomes, and a supportive institutional and health service framework [9]. Although many aspects of both technical and non-technical skills development can be achieved outside the operating room, the development and enhancement of certain skills still require learning in the operating room [2], where the types of learning models may be individualistic and determined by the learner’s competence and attitudes [10, 17].

Considering perception in the present study, both learners and teachers agreed that the appropriate learning loads for the objectives should increase gradually. However, Rose et al. reported there were significant differences between resident and faculty surgeons with respect to teaching the operative steps, surgical skills, instrument handling and surgical technique [17]. In the present study, in addition to significant disparity in learning and teaching between learner and teacher perceptions, junior and senior residents have different learning needs in the operating room even though both scrub in the same case. Cunha-Melo and Costa reported expectations and competencies of junior learners differ greatly from that of senior learners [19]. Park et al. reported resident perception of confidence differs from junior to senior residents [33]. Torbeck et al. reported more autonomy should be given to senior than to junior residents [34]. Consequently, an expert might better provide guided-discovery learning for the novice with preparatory information before the experience, and offer verbal and perhaps manual guidance when necessary, during the experience and feedback afterward [15].

Technical skills are important for successful surgical practice. Surgical technique was the learning preference from learner perspective; meanwhile, surgical teachers perceived that the higher the learner level, the higher priority set on the objectives needed. However, a traditional focus on the acquisition of technical skills and competence is no longer enough for the delivery of modern and safe surgical practice [9]. There is increasing evidence that patient harm is not due to deficient technical skills alone. Poor decision-making and deficiencies in teamwork are reported as contributors to adverse events and patient harm in the operating room. Therefore, non-technical skills are of increasing importance in surgery and surgical training [9]. Several assessment tools have been developed to evaluate learner non-technical performance [35, 36], and the most extensively used and validated training tool is the NOTSS behavior marker system, include situational awareness, decision-making, communication & teamwork, and leadership [25].

In the present study, learner perspectives on the non-technical skills such as clinical reasoning and teamwork were significantly different from those of the surgical teacher. The surgical teachers perceived that clinical reasoning was learning preference for SL. Although surgical teachers perceived that successful teamwork was learning preference for both JL and SL, the learners did not regard the learning objective as their main preference. In the operating room, surgeons should need the skill of evidence-based clinical reasoning to make the best real-time decision and work with a ‘well-oiled’ multidisciplinary team with clear pathways for patients, good handoffs and communication practices [8, 9, 37]. Therefore, the operation room is an excellent milieu providing opportunities to teach not only technical skills but also non-technical skills like clinical reasoning and teamwork.

Our profession is based on ethical behavior, which extends far beyond the rule of law [38]. Surgeons are often faced with the challenge of balancing truth-telling and the maintenance of hope in the setting of a poor prognosis or an adverse surgical event [25, 27]. Our study identified that both learners and surgical teachers agreed ethical issues were the learning preference for SL. It is necessary to understand how to communicate uncertainty and risk and how to make a disclosure to the family during or after an operation [27]. Several authors have suggested that training on disclosing adverse events using specific communication skills could be developed in the medical school curriculum [35, 39].

Several limitations in the present series has been identified. The current study recruited a relatively small number of learners and surgical teachers and examined practice at only one training center, so one could argue that it might not be reflective of all. Larger scale research on students and teacher perspectives at multi-medical centers is necessary. Since both learners and surgical teachers have expectations mutually agreed on, this would optimize performance of every participant and reduce barriers of communication while increasing confidence, which would eventually lead to better learning outcome and patient safety [2].

## Conclusions

Our study revealed significant disparity of perspectives between teachers and learners on perioperative teaching and learning. Resident perspective on learning objectives in the operating room differed between JL and SL even though both scrub in the same case, and there was discrepancy between resident’s learning as perceived by teachers, particularly in junior learners. Surgical teachers should set an appropriate quantity and quality of learning objectives for different learner levels. Effective communication between teachers and learners has the potential to improve learning experience and create a positive environment in the operating room.

## Supplementary information

**Additional file 1.**

## Data Availability

All data and materials are available.

## Abbreviations

PGY: Postgraduate year
JL: Junior learner
SL: Senior learner
