# Supplementary material for: Disparity of perspectives between teachers and learners on perioperative teaching and learning
Source: BMC Med Educ. 2020 Jul 31;20:244. doi: 10.1186/s12909-020-02172-8 (PMC7393732; doi:10.1186/s12909-020-02172-8)
Supplement: Supplementary file 1 — Additional file 1. [file 12909_2020_2172_MOESM1_ESM.zip › Appendix. Evaluation (Chinese Version)R2.docx]

**台灣手術室教學現況調查-教師觀點問卷**

第一部分：手術室的學習目標

1. 您認為資淺住院醫師 (PGY, R1 & R2)在手術室最重要的學習目標。(可複選)

□手術室術前準備；□解剖學；□手術適應症；□手術器械；□手術技術；□手術步驟；□獨自操作的能力；□手術中臨床推理能力；□手術併發症；

□團隊合作能力；□醫學倫理議題；□手術室術後的照護；□其他___________

2. 您認為資深住院醫師 (R3, R4, R5 & R6)在手術室最重要的學習目標。(可複選)

□手術室術前準備；□解剖學；□手術適應症；□手術器械；□手術技術；□手術步驟；□獨自操作的能力；□手術中臨床推理能力；□手術併發症；

□團隊合作能力；□醫學倫理議題；□手術室術後的照護；□其他___________

第二部分：基本資料

3. 性別：□男；□女

4. 年齡：□31-40歲；□41-50歲；□51-60歲；□61-65歲；□>65歲

5. 請問您現為主治醫師第____年。

6. 請問您具備的教職身分，□無；□講師；□助理教授；□副教授；□教授。

7. 請問您曾經獲得高醫附設醫院優良教學主治醫師，□無；□有，共____次。

8. 衛生福利部認定執業科別

□外科；□整形外科；□神經外科；□婦產科；□泌尿科；□骨科

9. 服務教學研究是高醫附設醫院的宗旨，請問您覺得教學在外科醫師忙碌的日常生活所佔的比例約為：

□0-20%； □21-40%； □41-60%； □61-80%； □81-100%。

**台灣手術室教學現況調查-學員觀點問卷**

第一部分：手術室的學習目標

1. 請問您認為目前在手術室最重要的學習目標？(可複選)

□手術室術前準備；□解剖學；□手術適應症；□手術器械；□手術技術；□手術步驟；□獨自操作的能力；□手術中臨床推理能力；□手術併發症；

□團隊合作能力；□醫學倫理議題；□手術室術後的照護；□其他___________

2. 現階段老師認為您在手術室最重要的學習目標？(可複選)

□手術室術前準備；□解剖學；□手術適應症；□手術器械；□手術技術；□手術步驟；□獨自操作的能力；□手術中臨床推理能力；□手術併發症；

□團隊合作能力；□醫學倫理議題；□手術室術後的照護；□其他___________

第二部分：基本資料

3. 性別：□男；□女

4. 年齡：□<25歲；□25-30歲；□31-40歲；□41-50歲

5. 臨床年資：

□PGY；□R 1；□R 2；□R 3；□R 4；□R 5；□R 6

6. 服務教學研究是高醫附設醫院的宗旨，請問您覺得教學在外科醫師的工作時數所佔的比例約為：

□0-20%；□21-40%；□41-60%；□61-80%；□81-100%。
