# Supplementary material for: Disparity of perspectives between teachers and learners on perioperative teaching and learning
Source: BMC Med Educ. 2020 Jul 31;20:244. doi: 10.1186/s12909-020-02172-8 (PMC7393732; doi:10.1186/s12909-020-02172-8)
Supplement: Supplementary file 1 — Additional file 1. [file 12909_2020_2172_MOESM1_ESM.zip › Appendix. Evaluation (English Version)R2.docx]

**Surgical Teacher Perspective in the Operating Room**

Part 1: Learning objectives in the operating room

1. What do you think are the most preferable learning objectives in the operating room for senior learners (R3, R4, R5 & R6)? (Multiple choices)

□ Preoperative preparation in the operating room

□ Surgical anatomy

□ Operative indications

□ Instrument handling

□ Surgical technique

□ Details of procedure (Verbally describing surgical procedures step-by-step)

□ Independent practice

□ Clinical reasoning

□ Complications

□ Teamwork

□ Ethical issues (medical errors, truth-telling, and disclosure)

□ Postoperative care in the operating room

□ Other_______________

2. What do you think are the most preferable learning objectives in the operating room for junior learners (PGY, R1 & R2)? (Multiple choices)

□ Preoperative preparation in the operating room

□ Surgical anatomy

□ Operative indications

□ Instrument handling

□ Surgical technique

□ Details of procedure (Verbally describing surgical procedures step-by-step)

□ Independent practice

□ Clinical reasoning

□ Complications

□ Teamwork

□ Ethical issues (medical errors, truth-telling, and disclosure)

□ Postoperative care in the operating room

□ Other_______________

Part 2: Basic information

3. Gender：□ Male　□ Female

4. Age：□ 31-40 yrs　□ 41-50 yrs　□ 51-60 yrs　□ 61-65 yrs　□ >65 yrs

5. What is your seniority as an attending physician? ______ Year

6. What is your faculty position at the medical school?

□ None　　　□ Lecturer　　　□ Assistant professor

□ Associate Professor　　　□ Professor

7. Have you ever won the “Annual Teaching Award” as an attending physician at Kaohsiung Medical University Hospital?

□ None　　　□ Yes, how many times? ___________

8. What is your specialty?

□ General Surgery　　　□ Plastic Surgery　　　□ Neurosurgery

□ Obstetrics and Gynecology　　　□ Urology　　　□ Orthopedics

9. What percentage of work hours do you think teaching occupies for surgeons?

□ 0-20%　　□ 21-40%　　□ 41-60%　　□ 61-80%　　□ 81-100%

**Learner Perspective in the Operating Room**

1. What is the most preferable learning objective you should learn in the operating room? (Multiple choices)

□ Preoperative preparation in the operating room

□ Surgical anatomy

□ Operative indications

□ Instrument handling

□ Surgical technique

□ Details of procedure (Verbally describing surgical procedures step-by-step)

□ Independent practice

□ Clinical reasoning

□ Complications

□ Teamwork

□ Ethical issues (medical errors, truth-telling, and disclosure)

□ Postoperative care in the operating room

□ Other_______________

2. What is the most preferable learning objective the teacher should teach in the operating room? (Multiple choices)

□ Preoperative preparation in the operating room

□ Surgical anatomy

□ Operative indications

□ Instrument handling

□ Surgical technique

□ Details of procedure (Verbally describing surgical procedures step-by-step)

□ Independent practice

□ Clinical reasoning

□ Complications

□ Teamwork

□ Ethical issues (medical errors, truth-telling, and disclosure)

□ Postoperative care in the operating room

□ Other_______________

Part 2: Basic information

3. Gender：□ Male　□ Female

4. Age：□ <25 yrs　□ 25-30 yrs　□ 31-40 yrs　□ 41-50 yrs

5. Clinical year：

□ PGY □ R 1　　□ R 2　　□ R 3　　□ R 4　　□ R 5　　□ R 6

6. The mission statement of KMUH is to serve, teach, and research. What percentage of a surgeon’s total work hours do you think he or she spends on teaching?

□ 0-20%　　□ 21-40%　　□ 41-60%　　□ 61-80%　　□ 81-100%
